# Heterologous Production of Flavour and Aroma Compounds in *Saccharomyces cerevisiae*

**DOI:** 10.3390/genes9070326

**Published:** 2018-06-28

**Authors:** Dariusz R. Kutyna, Anthony R. Borneman

**Affiliations:** The Australian Wine Research Institute, P.O. Box 197, Glen Osmond, SA 5064, Australia; darek.kutyna@awri.com.au

**Keywords:** synthetic biology, *Saccharomyces cerevisiae*, genetic engineering, heterologous production of flavour and aroma compounds

## Abstract

Over the last two decades, rapid progress in the field of synthetic biology has opened several avenues for the heterologous de novo production of complex biological compounds, such as biofuels, pharmaceuticals, and food additives in microbial hosts. This minireview addresses the usage of the yeast *Saccharomyces cerevisiae* as a microbial cell factory for the production of flavour and aroma compounds, thereby providing a path towards a sustainable and efficient means of producing what are normally rare, and often expensive plant-derived chemicals.

## 1. Introduction

Synthetic biology is one of the most rapidly evolving branches of the biological sciences. It allows the introduction of custom made genetic pathways into organisms that provide them the ability to display biological and/or biochemical properties over-and-above the original, wild type background. Numerous examples have been recently reported of the successful implementation of synthetic biology in microorganisms such as *Saccharomyces cerevisiae*, such as for the production of valuable biomedical compounds [1] or biofuels [2,3]. Many different microorganisms, like *Yarowia lippolitica* or *Escherichia coli*, have also been utilized as hosts for various synthetic biology applications [4,5]. However, because of the availability of a vast array of well-established molecular and genetic techniques, baker’s yeast *S. cerevisiae* continuously remains one of the most frequently used microorganisms in this rapidly growing field. In this minireview, attention will be focused on the utilization of *S. cerevisiae* for the heterologous production of flavour and aroma compounds, and their potential applications in food and beverage industries. 

## 2. Heterologous Compounds Derived from Intermediates of the Shikimate Pathway

**Vanillin**, (4-hydroxy-3-methoxybenzaldehyde), is a plant secondary metabolite commonly extracted from the seed pods of the vanilla orchids *Vanilla planifolia*, *Vanilla tahitensis*, or *Vanilla pompona*. It is one of the most commonly used food and beverage flavouring additives, with over 16,000 tonnes of vanillin consumed worldwide each year [6]. However, the slow growth of the vanilla orchid combined with the relatively low content of vanillin in the mature seed pods, means that only a minor fraction (~0.25%) of the total production of this compound is actually derived from vanilla orchids [7]. Market demand for this flavouring agent is therefore mostly fulfilled through chemical synthesis from lignin and/or fossil hydrocarbons, processes that are regarded as environmentally unfriendly and not sustainable. Alternative means of non-synthetic vanillin production are therefore highly desirable [8].

**Figure 1:**
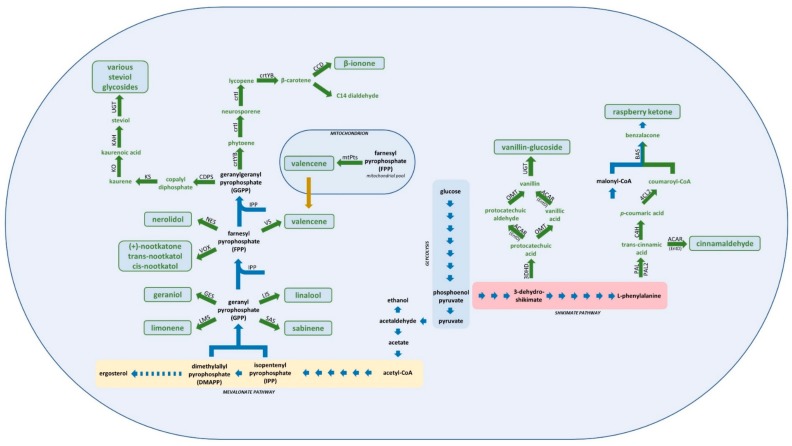
Simplified central metabolism of *Saccharomyces cerevisiae* (blue arrows), and heterologous enzymatic reactions (green arrows) leading to production of various flavour and aroma compounds: vanillin-glucoside (3DSD: 3-dehydroshikimate dehydratase, ACAR: aryl carboxylic acid reductase, OMT: *O*-methyltransferase, UGT: UDP-glycosyltransferase), raspberry ketone (PAL: phenylalanine/tyrosine ammonia lyase, C4H: cinnamate-4-hydroxlase, 4CL2: coumarate-CoA ligase 2, BAS: benzalacetone synthase), cinnamaldehycde (PAL2: phenylalanine ammonia lyase 2, ACAR: aryl carboxylic acid reductase, EntD: phosphopantetheinyl transferase), geraniol (GES: geraniol synthase), limonene (LMS: limonene synthase), linalool (LIS: linalool synthase), sabinene (SAS: sabinene synthase), valencene (VS: valencene synthase, mtPts: mitochondria targeted valencene synthase), (+)-nootkatone, *trans*-nootkatol, *cis*-nootkatol (VOX: (+)-valencene oxidase), nerolidol (NES: nerolidol synthase), β-ionone (crtYB: phytoene synthase/lycopene cyclase, crtI: phytoene desaturase, CCD: carotenoid cleavage dioxygenase), steviol glycosides (CDPS: copalyl diphosphate synthase, KS: kaurene synthase, KO: kaurene oxidase, KAH: kaurenoic acid-13-hydroxylase, UGT: UDP-glycosyltransferase).

Heterologous biosynthetic production of vanillin by *S. cerevisiae* is one of the most widely recognized examples of applying a synthetic biology system for the industrial production of a high value compound via a microbial cell factory. The metabolic pathway that allows yeast to produce vanillin was first developed nearly a decade ago, requiring the expression of four heterologous enzymes; 3-dehydroshikimate dehydratase (3DSD) from the dung mold *Podospora pauciseta*, an aromatic carboxylic acid reductase (ACAR) from a bacterium of the *Nocardia* genus, an *O*-methyltransferase from *Homo sapiens* (*Hs*OMT), and a phosphopantetheinyl transferase from *Corynebacterium glutamicum* (EntD); the latter enzyme was required for activation of ACAR via phosphopantetheinylation [9] (Figure 1). Introduction of these foreign genes provided *S. cerevisiae* with the ability to convert the endogenous metabolic precursor 3-dehydroshikimate (3-DHS), an intermediate of the shikimate pathway for aromatic amino acid biosynthesis, into vanillin at concentrations approaching 45 mg/L.

However, these initial attempts at optimizing the production of vanillin were complicated by the reported toxicity of vanillin against many microorganisms, including yeast, where concentrations exceeding 50 mg/L can significantly inhibit growth [9], thereby imposing a ceiling on maximum vanillin production levels. The problem of vanillin toxicity was solved via the heterologous expression of a glycosyltransferase (UGT) from *Arabidopsis thaliana*, which converted vanillin into its less toxic and more soluble form, vanillin-*b*-d-glucoside (VG). This strategy, along with flux engineering of the native yeast metabolism, involving removal of the pyruvate decarboxylase (*PDC1*) and glutamate dehydrogenase (*GDH1*) genes, as well as overexpression of glutamate dehydrogenase (*GDH2*), resulted in a vanillin production level of 500 mg/L. However, despite this significant increase, these levels were still far below the theoretical yields predicted through metabolic modelling analysis [7]. To remove further metabolic bottlenecks, two potentially rate limiting heterologous steps in the vanillin synthesis pathway were addressed by additional gene overexpression; ACAR, which converts protocatechuic acid (PAC) to protocatechuic aldehyde (PAL), and *Hs*OMT which catalyses the conversion of (PAL) to vanillin (Figure 1). While overexpression of ACAR had no effect on final VG production, a 30% improvement was observed in the *Hs*OMT overexpressing strain [10].

In an alternative study, Strucko and colleagues [11] investigated the impact of different yeast strains on the VG production. By reconstituting heterologous VG biosynthesis pathway in two commonly used yeast strains, *S. cerevisiae* CEN.PK and S288c, it was observed that the S288c background was able to produce up to a 10-fold greater concentration of VG under the same fermentation conditions. As there are hundreds of different strains of *S. cerevisiae* available, this work clearly demonstrates that the original genetic background is highly important in regards of choosing a yeast strain for an efficient microbial cell factory.

**Raspberry ketone** is the primary compound that contributes to the aroma of raspberries (*Rubus idaeus*) [12,13], although it is also produced by many other plants, including cranberries, blackberries, and rhubarb [14]. Raspberry ketone extracted from natural sources is an expensive flavoring agent, costing as much as US $3000/kg. However, as seen for vanillin, raspberry ketone can also be derived utilizing chemical synthesis, with the synthetic version costing far less than the naturally-derived version (US $58/kg) [15]. In *R. idaeus*, raspberry ketone is produced via the diketide pathway, starting with condensation of 4-coumaryl-CoA and malonyl-CoA by benzalacetone synthase (BAS) to form 4-(4-hydroxyphenyl)but-3-en-2-one (benzalacetone) [16,17]. In a subsequent reaction, benzalacetone is reduced by NADPH-dependent benzalactone reductase (BAR), also known as raspberry ketone/zingerone synthase 1 (*Ri*ZS1), to form raspberry ketone [18].

*S. cerevisiae* lacks the diketide pathway, with phenylalanine and tyrosine representing the metabolic branch points for heterologous de novo production of RK (Figure 1). Primary attempts of heterologous expression of plant genes, 4-coumarate-coenzyme A ligase (4CL) and chalcone synthase (CHS), in this yeast for raspberry ketone production were not entirely successful. The recombinant yeast grown in media containing *p*-coumaric acid produced only very low levels of raspberry ketone [19]. However, in a recent study, Lee et al. [14] engineered *S. cerevisiae* to express the complete de novo pathway for heterologous production of raspberry ketone, without necessity of supplementation with the precursor; *p*-coumaric acid. The recombinant yeast strain was genetically modified to produce *p*-coumaric acid through the expression of the heterologous enzymes phenylalanine/tyrosine ammonia lyase from *Rhodosporidium toruloides* (*Rt*PAL) (which provided both phenylalanine and tyrosine ammonia lyase activists), and cinnamate-4-hydroxylase from *A. thaliana* (*At*C4H), a combination shown previously to drive *p*-coumaric acid production in *S. cerevisiae* [20,21]. Additional expression of a synthetic fusion protein that linked coumarate-CoA ligase 2 from *Petroselinum crispum* and benzalacetone synthase from *Rheum palmatum* (*Pc*4CL2-*Rp*BAS), yielded a strain able to produce 3.5 mg/L of raspberry ketone [14]. Although levels of this highly valuable flavouring compound produced by the above recombinant strain are not yet adequate for its industrial application, this study paves the way for future development of *S. cerevisiae* cell factories for commercial raspberry ketone production.

**Cinnamaldehyde** is the main impact flavonoid found in the bark, lives and fruit of the cinnamon tree *Cinnamomum zeylanicum* [22,23]. It is the main organic compound that contributes the flavour and aroma of cinnamon and is commonly used as a flavouring compound in the food and beverage industries [24,25]. Many chemical methods exist to produce this flavonoid synthetically, however steam distillation of the oil derived from the cinnamon bark remains the most economical and commonly used method [26,27]. Cinnamaldehyde can be heterologously produced in *S. cerevisiae* through an extension of the shikimate pathway (via phenylalanine), through the activity of two heterologous enzymes; phenylalanine ammonia lyase 2 (PAL2) which converts phenylalanine into *trans*-cinnamic acid, and aryl carboxylic acid reductase (ACAR) which converts *trans*-cinnamic acid into cinnamaldehyde (Figure 1). As also seen for the production of vanillin, activation of ACAR was achieved by overexpression of a phosphopantetheinyl transferase [28]. Although successfully achieving a proof of concept for de novo production of cinnamaldehyde, only negligible amounts of this compound were produced, primarily due to a combination of cinnamaldehyde toxicity (~0.68 mM in *S. cerevisiae*) and off-target metabolism towards cinnamyl alcohol and hydrocinnamyl alcohol [28,29]. Thus, while heterologous production of cinnamaldehyde has been achieved, the transition to an industrial scale will require further metabolic optimization.

## 3. Heterologous Compounds Derived from Intermediates of the Mevalonate Pathway

### 3.1. Isoprenoids

Isoprenoids (also known as terpenes or terpenoids) are a large group of diverse organic compounds that encompass thousands of different chemical forms and which are mainly observed as the products of plants. Despite their chemical diversity, all isoprenoids are biosynthesized from the basic unit isoprene (C_5_H_8_), with different subclasses of compounds classified according to the number of isoprene units (hemiterpenes, monoterpenes, sesquiterpenes, and diterpenes with 1, 2, 3, and 4 isoprene units, respectively). Many of the compounds belonging to the terpene group are toxic and/or display unpleasant, irritant odours and are used by host plants to deter predatorial foraging and pathogen invasion [30]. However, a small selection of specific isoprenoinds have found application in variety of industries, including food and beverage as attractive flavouring additives.

#### 3.1.1. Monoterpenes

Monoterpenes are all produced via the action of monoterpene synthases acting on geranyl pyrophosphate (GPP) as a common substrate. Each individual monoterpene synthase is therefore responsible for producing a specific monoterpene (or a combination of monoterpenes) from this common metabolic precursor [31].

**Geraniol** is one of the most in-demand monoterpenes, primarily due to its pleasant rose-like aroma. It is now an established additive in the food and beverage industries, where it is commonly used to enhance the flavours of a broad range of beverages, ice cream, candy, chewing gum, and many other products [32].

Due to its high sensory impact, geraniol is produced in negligible concentrations in most plants, such that the yields of final extracted compounds are very low [33]. While plant sources of geraniol are generally limiting, yeast has proven to be a very efficient cell factory for the heterologous production of this compound. *S. cerevisiae* lacks the ability to produce monoterpenes, however it produces the penultimate monoterpene precursor, GPP, as part of the mevalonate/ergosterol pathway [34,35] (Figure 1). Interestingly, despite lacking recognised monoterpene synthase activity, *S. cerevisiae* strains carrying mutations in farnesyl diphosphate synthase gene (*ERG20*) were shown to produce low levels of geraniol (1.3 mg/L), presumably through non-specific production due to high GPP levels [36]. Later, Iijima and colleagues isolated and characterized the specific geraniol synthase (GES) from sweet basil (*Ocimum basilicum*) that catalyses conversion of GPP to geraniol [37], unlocking the possibility of geraniol production in microbial hosts (Figure 1). Heterologous expression of this enzyme in *S. cerevisiae* allowed production of geraniol at levels up to 500 µg/L in the growth medium [34]. Following on from this, engineering of ERG20p via amino acid substitutions surrounding the catalytic site resulted in a strain able to produce around 5 mg/L of geraniol [38].

Geraniol synthase enzymes have now been identified and characterized from a large number of different plant sources [39,40]. This expanded catalogue of natural variation, combined with additional metabolic engineering, has been used to further improve heterologous geraniol production in *S. cerevisiae*. By introducing a fusion protein composed of geraniol synthase from *Valeriana officinalis* (t*Vo*GES) that lacks plastid targeting signal and a mutant farnesyl diphosphate synthase ERG20p^(F96W-N127W)^, in a strain with a metabolically-engineered high-flux mevalonate pathway, 239 mg/L of geraniol was achieved in fed-batch cultures [41,42,43]. However, this concentration was still deemed inadequate for industrialization of the system. In an elegant study Jiang and colleagues [44] have now achieved much higher levels of geraniol production through a combination bioprospecting for highly active GES proteins, site directed mutagenesis and enzyme structural analysis, which ultimately resulted in a strain able to produce over 0.5 g/L of geraniol. When this strain was further tested in fed-batch fermentation under carbon restricted conditions, it produced 1.68 g/L of geraniol, the highest level of this monoterpene produced by any eukaryotic organism reported to date [44].

In parallel to approaches that seek to produce the highest amounts of geraniol possible for purification as an additive, alternative studies have been conducted to investigate the in situ production of geraniol during wine fermentation [45]. Expression of GES from sweet basil resulted in the accumulation of more than 750 µg/L of geraniol in finished wine that was made from aromatically neutral grapes. Surprisingly, in additional to geraniol, the final wine also contained significant amounts of additional monoterpenes, including citronellol, linalool, and nerol, which were likely derived from indigenous enzymatic activities in the industrial wine yeast background. Although the amounts of geraniol produced by this recombinant industrial yeast were much lower compared to the heterologous systems described above, the levels of this, as well as other monoterpenes, were way above the olfactory sensory thresholds, positively contributing to overall sensory characteristics of wines by providing enhanced floral and fruity attributes [45].

**Linalool** can be naturally found in many fruits and flowers and is responsible for pleasant floral aromas. Heterologous production of this flavour compound in yeast was originally achieved via the expression of a linalool synthase (LIS) from *Clarkia breweri*, one of the earliest identified monoterpene synthases, and which specifically converts GPP to linalool [46,47] (Figure 1). While initial concentrations of linalool were very low (~22 µg/L), engineering of flux through the mevalonate pathway via overexpression of catalytic domain of Hmg1p (3-hydroxy-3-methylglutaryl coenzyme A reductase), combined with specific yeast strain selection, resulted in a 6-fold increase in linalool concentrations [48]. A subsequent study that used LIS from *Lavandula angustifolia*, combined with overexpression of *HMG1* and downregulation of *ERG9*, resulted in levels of up to 95 µg/L of linalool [49]. However, yields remain well behind those observed for geraniol and further optimization of this system is required if industrially-relevant production levels are to be achieved.

As for geraniol, there have also been attempts to produce linalool in situ during fermentation instead of as a purified additive. Linalool and geraniol are the main impact molecules that confer dry-hopped flavour and aroma in specific styles of beer. In classical brewing, these monoterpenes originate from the flowers of the hop plant, which are added to the wort during fermentation. In a recent study, Denby et al. [50] screened for combination of linalool and geraniol synthases and gene promoters, that could be combined in brewing yeast to produce these monoterpenes at levels comparable to those achieved by standard dry-hopping during the beer fermentation process. A brewing strain of *S. cerevisiae*, that was metabolically engineered to produce higher levels of GPP, was equipped with a truncated LIS from *Mentha citrata* and full-length GES from *Ocimum basilicum,* yielding a self-flavouring brewing yeast able to produce beer with basic dry-hopped flavour profiles, without the necessity of adding hop flowers.

**Limonene** is another example of terpenoid that is commonly used as a flavouring agent [25]. It is mainly found in citrus fruit peels and used in food and beverage products such as candy and soft drinks [51]. With an annual production of over 60,000 tonnes [52], it is primarily obtained from citrus essential oils, which are byproducts from orange juice production, and may contain over 90% of this compound in the total pool of the compounds present [53,54]. As the price of natural limonene is relatively high, its production in microbial hosts would reduce the dependence on its industrial production from plant sources, such as citrus fruits.

The potential for heterologous production of limonene in *S. cerevisiae* has recently been reported, where it is based on the same metabolic principals as those for the production of geraniol, except for the final step in which GPP is converted to limonene by a limonene synthase (LMS) (Figure 1). However, the reported yields of limonene remain far lower than those of either geraniol or linanool, with a maximum of 0.12 mg/L (+)-limonene and 0.49 mg/L (−)-limonene produced in *S. cerevisiae* [55,56]. These low production levels suggest that major metabolic redesign and optimization are required for limonene production to be industrially applicable. However, as 27 limonene synthases, derived from 9 different plant families, have recently been characterized [57], this opens new possibilities for metabolic engineering and/or enzyme optimization in yeast, with potential to improve the yields of this valuable monoterpene.

**Sabinene** is another example of monoterpene that was reported to be produced in *S. cerevisiae* by means of heterologous gene expression. This monoterpene can be naturally found and extracted from the essential oils of many plants, including *Hyptis pectinata* Poit. (Lamiaceae) [58], or *Zornia diphylla* (L.) Pers, where it can reach more than 40% of the total oil extracted from whole plant [59]. In *S. cerevisiae* production of sabinene was achieved by heterologous expression of highly specific sabinene synthase (SAS) from *Salvia pomifera* [60] (Figure 1). By introduction of fusion protein ERG20p^(F96W-N127W)^-*Sp*SASp nearly 1.9 mg/L of sabinene was produced by this recombinant yeast. Subsequent deletion of one copy of the wild type ERG20, and introduction of the second copy of fusion protein ERG20p^(F96W-N127W)^-*Sp*SabS1 further improved sabinene production to 17.5 mg/L.

#### 3.1.2. Sesquiterpenes

Sesquiterpenes are a diverse group of plant secondary metabolites which are generally derived from C-15 precursor; farnesyl pyrophosphate (FPP).

**Valencene** can be found in many citrus fruits [61,62] and is commonly and inexpensively extracted from the essential oil of Valencia oranges. Heterologous production of this sesquiterpene in *S. cerevisiae* was achieved by identification and expression of valencene synthase (VS), with variants of this enzyme having been characterized from many plants including *Citrus paradisi* (grapefruit), *Citrus sinensis* or Nootka cypress (*Callitropsis nootkatensis*) [63,64]. Expression of the *C. paradisi* VS in a *S. cerevisiae* strain (Figure 1), which was metabolically engineered to produce elevated levels of FPP [65], was shown to produce low levels of valencene (5.3 µg/L) [66].

Later, Farhi and colleagues [63] have altered metabolic flux through the mevalonate pathway for enhanced farnesyl pyrophosphate (FPP) accumulation by overexpression of tHMG in *C. sinensis* valencene synthase *CsTPS1* expressing yeast strain, which led to 1.5-fold (ca. 40 to ca. 60 µg/L) increase in valencene production, compared to the expression of *CsTPS1* alone. Introduction either *A. thaliana* short isoform of farnesyl diphosphate synthase (*At*FDPS), or *H. sapiens* farnesyl diphosphate synthase (*Hs*FDPS), further increased valencene production; the final strain carrying *CsTPS1*, *tHMG* and *AtFDPS* was able to produce 370 µg/L of valencene.

Subsequent investigation of the effects of cellular compartmentalization have led to identification of the mitochondrion as a potential sub-cellular location for terpene production. To achieve this, the authors equipped *CsTPS1* with mitochondrial targeting peptide from the *COX4* gene, studied earlier by Hurt et al. [67], to test whether mitochondrial pool of FPP can be utilized for valencene production. Expression of mitochondrial *CsTPS1 (mtCsTPS1*) by itself resulted in nearly 3-fold increase in valencene production, followed by further 50% increase when co-expressed with *tHMG*, and additional 40% increase while mitochondria targeted FDPS (*mt*FDPS) was used, yielding ca. 1.2 mg/L of valencene. Ultimately, the expression of an additional cytosolic copy of *CsTPS1* resulted in production levels of approximately 1.5 mg/L of this sesquiterpene, suggesting that both mitochondrial and cytosolic pools of FPP might be used for the production of terpenes.

In a recent study, the utility of exploring natural protein variation was highlighted by the discovery of an alternative valencene synthase derived from the heartwood of the Nootka cypress (CnVS) [64]. In comparison to valencene synthase obtained from *C. paradisi*, expression of CnVS in a non-optimized *S. cerevisiae* strain produced 1.36 mg/L of valencene, an almost 450-fold increase in production compared to the citrus equivalent and a level which approximates those achieved via extensive metabolic optimization of strains containing the citrus enzymes. This new valencene synthase may therefore represent a valuable resource for further optimization and industrial scale production of this sesquiterpene.

**(+)-nootkatone** is an oxidized form of the sesquiterpene (+)-valencene, with a characteristic grapefruit flavour and relatively low sensory threshold [68]. It is found naturally in (and can be extracted from) plants including Citrus (*Rutaceae* spp.), Java (*Cyperus rotundus*), and Vetiver grass (*Vetiveria* spp.) [69,70]. Due to the very low concentrations of nootkatone found in natural sources, a synthetic compound is currently used for industrial applications [71]. However, as seen for many other flavour and aroma compounds, this synthetic production usually involves environmentally unfriendly processes and chemicals so heterologous production sources are being investigated [72,73].

Conversion of (+)-valencene to its oxidation products; (+)-nootkatone and α- and β-nootkatole (all having pleasant grapefruit-like aromas) are thought to be catalysed by members of the cytochrome P450 monoxygenase superfamily. Co-expression of the P450 enzyme CYP71AV8 from chicory root (*Cichorium intybus*), also known as (+)-valencene oxidase (VOX), which was reported to convert valencene to nootkatone via its oxidation at the C2 positions, along with valencene synthase from *C. sinensis*, allowed a recombinant *S. cerevisiae* strain to convert 68% of total pool of (+)-valencene to *trans*-nootkatol (0.92 mg/L), 8% to *cis*-nootkatol (0.11 mg/L), and 3% to (+)-nootkatone (0.04 mg/L) [74] (Figure 1).

In a later study, Gaviera et al. [75] used an in silico gene mining approach to identify and characterize novel cytochrome P450 valencene 2-oxygenases; CYP71D4 from *Solanum tuberosum*, CYP71D51v2 from *Nicotiana tabacum*, CYP71D1 from *Catharanthus roseus*, and CYP71D326 from *Ricinus communis*. Subsequently, they tested each of these oxygenases by introducing them into WAT11 yeast strain, which was genetically modified for stable expression of *A. thaliana* P450 reductase *ATR1* [76]. The tobacco (*Nicotiana tabacum*) enzyme, CYP71D51v2, displayed the highest expression levels in this recombinant yeast, as well as efficient conversion of (+)-valencene to β-nootkatol and low levels of undesired byproducts. Incubation of this yeast strain with increasing (+)-valencene concentrations resulted in maximum combined yield of 6 mg/L of β-nootkatol and (+)-nootkatone, while 200 mg/L (+)-valencene was used. Further increase of (+)-valencene feed concentrations resulted in lower conversion rates, likely due to increased substrate and/or product toxicity.

Production of *trans*-nootkatol was further improved by overexpression of the native yeast type III membrane associate protein Ice2p [77]. Introduction of this genetic modification into recombinant strain engineered earlier to produce (+)-valencene via expression of premnaspirodiene oxygenase (HPO; CYP) from *Hyoscyamus muticus* and cytochrome P450 from *A. thaliana* (*At*CPR) [64], resulted in stabilizing the levels and enzymatic activity of the latter heterologous enzyme. This modified recombinant strain was able to synthesize approximately 30 mg/L of *trans*-nootkatol. Although promising, the heterologous nootkatone production system would need to be further improved for its potential application on the industrial scale.

**Nerolidol** occurs in a broad spectrum of plants and, in its purified form, displays wood- and fresh bark-like aromas. It is officially permitted to be used as food flavouring additive by the U.S. Food and Drug Administration (FDA) and is currently used in industrial processing [25].

Recently, a study has explored the heterologous production of this sesquiterpene by *S. cerevisiae* via the expression of a nerolidol synthase (NES) from *Actinida chinensis* in an *ERG20* overexpression background, resulting in the production of nerolidol in the growth media at 5 mg/L [78]. Further pathway optimization of this strain, which included replacing the initial three enzymes of “upper” MEV pathway (*ERG10*, *ERG13*, and *tHMG1*) with codon optimized heterologous counterparts and co-expressing HMG-CoA synthase and a bi-functional acetoacetyl-CoA thiolase/HMG-CoA reductase from *Enterococcus faecalis* resulting in over a ten-fold increase in nerolidol (56 mg/L). The authors also addressed the issue of undesired production of high levels of squalene, resulting from enzymatic activity of squalene epoxidase (*ERG9*), which competes with the heterologous nerolidol synthase (*Ac*NES1) for the cellular pools of FPP. As production of squalene is essential for the survival of yeast, they implemented elegant system in which the degradation of the native Erg9 protein was enhanced, thereby decreasing its cellular half-life and enzymatic activity and increasing the production of *trans*-nerolidol by 86% (105 mg/L) [79,80].

#### 3.1.3. Norisoprenoids

Norisoprenoids are a group of C-13-carotenoid-derived aromatic compounds, produced by many fruit and flowers, such as grapes, peaches, blackberries, and which are greatly valued as flavouring additives [81]. In plants, norisoprenoids are produced by the enzymatic activity of carotenoid cleavage oxygenases (CCO), which cleave carotenoids at positions C9 through C13 and thereby yielding different classes of these compounds [82].

**β-ionone** is one of the most common of this group of aromatic compounds, displaying floral, violet-like characteristics and exhibiting an extremally low odour threshold of 0.007 ppb (in water) [81]. Due to its desirable properties β-ionone has been used as a food and beverage additive to enhance the flavour of ice cream, candy, baked goods, gelatines, puddings, chewing gum, and non-alcoholic beverages [83].

While β-ionone can be extracted from plants, where it is naturally produced by the enzymatic cleavage of β-carotene by carotenoid cleavage dioxygenase (CCD1), the overall process is tedious and expensive, and highly depends on availability of agricultural resources. Thus, microbial heterologous production of this valuable compound is an interesting alternative. De novo biosynthesis of β-carotene in S. cerevisiae, which is a precursor for β-ionone production, were successfully achieved by expression of three heterologous genes from the ascomycete *Xanthophyllomyces dendrorhous*; phytoene desaturase (crtI), GGPP synthase (crtE), and phytoene synthase/lycopene cyclase (crtYB) [84] (Figure 1). Later, Beekwilder and colleagues [85] used the same enzymes, along with expression of carotenoid-cleavage dioxygenase from raspberry (RiCCD1), demonstrating the possibility of heterologous production of β-ionone in *S. cerevisiae*. Relatively low amounts (0.22 mg/L) of this compound were produced by the recombinant yeast, which was presumably associated with the low efficiency of translation of the single polycistronic episomal construct carrying *crtYB*/*crtI*/*crtE* genes. Recently, Lopes et al. [86] reported engineering a more efficient heterologous strain of *S. cerevisiae* that was able to produce more than 5 mg/L of β-ionone. It was achieved by genetically modifying previously reported, FPP overproducing strain (SCGIS22) [87], via overexpression of *tHMG1* and GGPP synthase gene *BTS1*, along with expression of heterologous crtI and crtYB from X. dendrorhous, and CCD1 from *Petunia hybrida* (*PhCCD1*) (Figure 1)

## 4. Next Generation Sweeteners

Excessive consumption levels of high-caloric sweeteners, such as sugars, has been proven to have multiple negative impacts on human health, resulting in gain of weight, high blood pressure, or type 2 diabetes [88]. Thus, there is growing demand in food and beverage industries for a ‘new generation’ sweetening agents, which would provide to the consumers similar to common sugars perception of sweetness, while significantly restricting the high calorie intake. Natural plant derived compounds, such as stevia glucosides, has proven to be an attractive solution.

### Steviol Glycosides

Steviol glycosides (SGs) are a group of active compounds found in the leaves of *Stevia rebaudiana* Bertoni (also known as “sweet herb”), which has been used for centuries as a natural sweetener by native people of South America [89]. The SGs present in stevia plants; stevioside (Ste), rebaudioside- (Reb-) A, B, C, D, E, F, and M, dulcoside A, steviolbioside, and rubusoside, have distinctive sweet tastes that are estimated to range from 30 to 250 times more potent than sucrose [90]. Given these properties, it is not surprising that several SGs are now being used in the food and beverages industries as alternative, low-caloric sweetening agents [91].

The natural biosynthesis of SGs involves the isoprenoid pathway, with GGPP representing the metabolic branch point from this core metabolic pathway [92]. Four novel enzymatic steps then lead to the production of kaurenoic acid, which is further converted to steviol by kaurenoic acid hydroxylase (KAH). From this point, the activity of a variety of specific UDP-glycosyltransferases (UGTs) on the basic steviol molecule gives rise to the various SGs that are observed in plant extracts. The above metabolic steps were used to introduce de novo SGs production pathway into *S. cerevisiae* with five heterologous enzymes driving production of steviol from GGPP (Figure 1).

Fortunately, the heterologous production of SGs from steviol is aided in yeast by the presence of naturally high levels of cytosolic UDP-glucose [93]. This intracellular UDP-glucose pool can therefore be exploited as a sugar-donor by various heterologously-expressed UDP-glucosyltransferases for the synthesis of specific SGs. Efficient bioconversion (60%) of supplemented stevioside (relative sweetness of ~210) to rebaudioside A (relative sweetness of ~200), has been recently achieved by expressing the UDP-glycosyltransferase, UGT76G1, from *S. rebaudiana* in *S. cerevisiae* [94].

However, complicating the production of specific SGs is the fact that many glucosyltransferases display loosely-constrained substrate requirements and have the ability to catalyze multiple different glycosylation reactions. This leads to the co-synthesis of mixtures of different SGs, rather than the production of a single pure compound. For example, UGT76G1, which catalyzes the formation of highly desirable steviol glucosides, such as rebaudioside M and D, is also shown to produce undesirable byproducts, including rebaudioside G, Q, I and 1,3-bioside [90]. To address this enzymatic promiscuity, Olsson and colleagues [95] used protein site saturation mutagenesis to produce variants in both the predicted substrate-binding pocket, and enzyme active site UGT76G1. A library of 1748 mutants were screened using a strain of *S. cerevisiae* that carried the set of heterologous genes required for generation of rebaudioside, but lucking a glycosyltransferase [96]. Several mutants, such as UGT76G1^Thr146Gly^ and UGT76G1^His155Leu^, displayed the desired increase in rebaudioside M and/or D production with minimal byproduct formation. The study brings important insights into the possibility of altering the enzymatic specificity of steviol glucosyltransferases towards more efficient production of desired SGs.

## 5. Conclusions

The estimated turnover of the flavour and fragrance industries has been estimated to reach over US $30 billion in 2017, with a compounding annual growth rate of 5.6% between 2012 and 2017 [97]. To fulfil the demands of this constantly growing market, chemical synthesis of flavour and aroma compounds has routinely been used. However, the processes involved are generally not sustainable and the final products are considered of synthetic origin, which dramatically decreases their market value. While extraction of flavours and fragrances from natural sources provides a more valuable, natural product, there are number of limiting factors that make these processes inefficient and relatively expensive, including the availability of the source plant materials, and the low levels of the desired compounds in planta. Thus, there is a constantly growing demand for new, improved biotechnologies which allow more efficient, cheaper, and environmentally friendly ways to produce aroma and flavour compounds.

Over the last two decades, the rapidly growing field of synthetic biology has provided the possibility for the heterologous production of specific flavours and aromas in microorganisms, a unique resource that could be used to help fulfil these global demands. The biotechnological company Evolva [98] has taken advantage of synthetic biology and yeast for the industrial scale heterologous production of natural flavour and aroma compounds, such as nootkatone, valencene, vanillin, and next-generation sweeteners. However, from the thousands of flavour and aroma compounds that have been identified to date, only a limited number have been successfully produced by heterologous means. Along with new advances in metabolic engineering of *S. cerevisiae* [99], this presents an opportunity for development and potential industrial applications of new heterologous pathways to produce novel valuable compounds in yeast.
